# An evaluation of the EASY instrument in a cross-sectional study

**DOI:** 10.1186/s12874-024-02158-w

**Published:** 2024-02-10

**Authors:** Julie Agel, Umesh Ghimire, Nicholas M. Edwards, Bradley Nelson, Todd Rockwood

**Affiliations:** 1 Department of Orthopedics, University of Minnesota, 2450 Riverside Ave, Minneapolis, Minnesota USA; 2 Division of Health Policy and Management, University of Minnesota School of Public Health, 420 Delaware St SE, Minneapolis, Minnesota USA

**Keywords:** EASY, Youth, Physical activity, Survey, Scoring methodology, Evaluation of activity surveys in youth

## Abstract

**Background:**

The purpose of this paper is to evaluate the impact of modifying the published scoring system to address identified potential weaknesses in the published scoring system for the Evaluation of Activity Surveys in Youth (EASY). A secondary purpose was to evaluate the EASY on children in Grades 1–5. The EASY is a self-report physical activity instrument for youth.

**Methods:**

Original EASY survey results were collected at one time point from an online panel from participants across the United States as part of a larger cross-sectional University of Minnesota project looking at children’s specific activity and sports participation between June and August 2019. Data was evaluated using three common scoring methods: simple summation, mean, and transformed summation. Data was compared by Grades 1–5 and 6–8.

**Results:**

The summary statistics of the scores show that there is no statistically significant difference across the scoring methods by population. A paired t-test evaluation of the different scoring methods shows that while the scores are very similar within methodology (simple summation, mean, transformed sum) they are all statistically significantly different from one another, which demonstrates that for any given individual the specific scoring methodology used can result in meaningful differences. The transformed sum provided the strongest methodologic result. Analysis also concluded that administering the scale by proxy to children from grades 1–5 resulted in similar responses to those in Grades 6–8 broadening the appropriate populations able to use this scale.

**Conclusion:**

The transformed sum is the preferred scoring method.

**Trial registration:**

Not applicable.

## Background

Physical activity is an integral part of human life that confers a multitude of benefits. Regular physical activities are essential to physical growth, to fulfill exercise needs, reduce the risk of excessive weight gain and maintain good mental health [1–3]. It is recommended that youth engage in 60 min of moderate-to-vigorous physical activity (MVPA) daily [4]. Physical Activity Guidelines for Americans published by the U.S. Department of Health and Human Services also recommends most of the exercise being aerobic in nature and supplemented with muscle strengthening and bone-strengthening [5].

An estimate from the U.S. Department of Health and Human Services says that 80% or more American adolescents do not meet recommended physical activity guidelines [5]. Public health agencies around the world endorse physical activity guidelines for children and update their recommendations based on findings from studies conducted in children at state and national levels [6]. Consortiums around the world have used different instruments and surveillance systems to track physical activity levels across different age categories. In 2001, the World Health Organization launched a standardized survey entitled the Global School-based Student Health Survey recognizing the importance of physical activity in children [7]. Surveys like the Youth Risk Behavior Survey (YRBS) have been regularly measuring physical activity in the United States [8].

The Evaluation of Activity Surveys in Youth (EASY) instrument was developed and evaluated in middle school students for use in grade and middle school age children [9]. The EASY instrument was compiled from a study conducted during 2012–2015 to assess children’s physical activity and is composed of 14-items that relate to different physical activities at school and home settings. The 14-items were evaluated using Rasch analysis and traditional correlational methods with reported sensitivity and specificity of 0.90 and 0.44, respectively [9]. The EASY instrument was designed to collect information that is inclusive of children’s general day-to-day activities over a seven-day recall period.

In accordance with the EASY scale design responses for all 14 activities were recorded as yes/no followed by recall of the number of days of participation (1–7 days) for those where the response was yes. The published scoring of the EASY instrument uses simple summative scoring; in which an initial ‘no’ is coded as a zero and then the number of days identified in the follow-up questions are summed to identify a final score with a maximum of 98.

Out of 14 items, 4 items are directly related to school activities; have PE/gym classes, play an organized school sports team, walk, or bike to or from school, and play actively during recess or other free time at school. The published scoring system for the school items allowed for up to 7 days of participation for the school-based items with no accommodation identified for the inclusion or exclusion of the school-based items regardless of whether a child is likely to be in school (i.e., administered during school breaks). In our study the use of the EASY created concerns about differentiating between true responses and respondent error creating an inaccurate estimation of children’s physical activity. The primary purpose of this paper is to evaluate the impact of modifying the published scoring system to address identified potential weaknesses in the published scoring system for the EASY. The secondary purpose was to evaluate the use of the EASY in Grades 1–5 as recommended by the original authors. The data for this secondary analysis is taken from responses received between June and August 2019 from a cross-sectional online panel with participants across the United States as part of a larger project looking at children’s specific activity and sports participation.

## Methods

### Study design

The respondents were obtained through an online panel by Dynanet (Columbia, MD). Data was collected between June 20, 2019, and August 19, 2019, as part of a longitudinal survey evaluation measuring youth sports participation. The first survey data contained the EASY, our variable of interest, and thus this was a cross-sectional collection of data at one point in time. A quota was imposed on the sample in which equivalent numbers of females and males were identified for each grade (1–12). The study utilized parental proxy report for each child. 977 respondents completed the survey. The analysis presented here is based on 303 children in grades 6 to 8 (a replication of the original EASY study grades for the population) and 311 children in grades 1 to 5 (a reflection of our target population by grades and a recommended study population by Pate et al.) [9]. As a function of the online panel answers to all questions were required thus there is no missing data.

Data was evaluated using three common scoring methods: simple summation, mean, and transformed summation. The simple summation was just the straight sum of responses 0–7 questions for a range of 0–98. The mean score is the average of all available individual question responses with a range of 0–7. The transformed sum is calculated using the following method: ((∑answers-absolute minimum of answered items)/range of answered items)*100; individualized by patient responses with a range of 0-100. Given that data from the online panel was collected in the summer many, if not most, of the respondents should not have answered the school items with anything more than a 0 thus we imposed an additional condition for analysis to reflect the 5 day versus 7 day responses.

Within each scoring method we have used the range of response to be either 0–7 days (original method) or the four questions tied to school activities recoded to a range of 0–5 (reflecting a traditional school week). Conditional scoring was evaluated for each method as well. If the respondent indicated that their child did none of the activities and the date of the survey was outside of the traditional school year, then the 4 school related items were not included in calculating the score. The three scoring methods treat missing data differently and thus, the omission of the school items, will affect the simple summation scoring, but will not affect mean / transformed sum scoring. These later two scoring methods both account for the impact of missing data (i.e. Mean = sum of answered items/total possible for answered items; likewise for transformed sum the minimum possible value and range exclude items with missing values from the determination of their values.

ANOVA was used to compare the effects of the scoring methods and the different population constraints.

The sample size for this project was based on estimates to evaluate an instrument for a larger study, a sub-set of the data is being used post-hoc to illustrate scoring issues associated with a measure of child activity (EASY).

Any bias in the respondents’ answers would be represented equally across all of the scoring methods evaluated in this work and not addressable.

## Results

To assess the magnitude of the children with responses outside the theoretical acceptable range the percentage of children who reported they did not participate in the school related activity over the past week, or participated 6 to 7 days a week are shown in Table 1. For Q1: Have PE/GYM and Q5: After school program 44–49% of responses indicated that the child did not participate in the activity regardless of school grade. For Q4: Active during recess/free time at school 28% (1st -5th ) and 35% (6th -8th ) reported no participation over the 7-day recall period. Q3: Walk or bike to school demonstrated 66–74% of responses reporting no participation. <= 10% of the study population reported 6 or 7 days a week for school related activities. < 3% of respondents reported that their children engaged in all 4 school related activities 6 or 7 days (1% for 1st -5th and 2% for 6th to 8th ).

**Table 1:** Percentage with no participation 6–7 days/week of participation on school related questions

	No Participation			6 of 7 Days a week		
Item	1st to 5th	6th to 8th	Overall	1st to 5th	6th to 8th	Overall
Q1: Have PE/GYM	153 (51%)	148 (49%)	301 (49%)	12 (4%)	18 (6%)	30 (5%)
Q3: Walk or bike to school	229 (74%)	(201) 66%	430 (70%)	9 (3%)	17 (6%)	26 (4%)
Q4: Active during recess/free time at school	86(28%)	107 (35%)	109 (31%)	32 (10%)	30 (10%)	62 (10%)
Q5: After school program	151 (49%)	134 (44%)	285 (46%)	17 (5%)	20 (7%)	37 (6%)

To confirm that we could combine the two grade groups a comparison of scores was done for each of the 3 scoring methods using both the original 7 point scale and the 5 point scale reflecting that most schools only meet 5 days a week. (Table 2)

**Table 2:** A comparison between grade groups across scoring methods by 7 days and 5 days

	Simple Sum (X, std)	Mean Scoring (X, std)	Transformed (X, std)
**7 days**			
Grade 1- 5 *N*=311	32, 16.8	2.3, 1.2	32.6, 17.2
Grade 6-8 *N* = 303	29.7, 18.2	2.1, 1.3	30.3, 18.7
	*P* = 0.10	*P* = 0.11	*P*=0.11
**5 days**			
Grade 1- 5 *N*=311	31.6, 16.1	2.3, 1.2	35.1, 17.9
Grade 6-8 *N* = 303	29.2, 17.6	2.1, 1.3	32.5, 19.5
	*P* = 0.08	*P* = 0.09	*P*=0.09

Statistical values are based on 5 decimal points by computer analysis versus 1 decimal point used here in the table) results from mean score differ based on the number of decimal points used

**Fig. 1:**
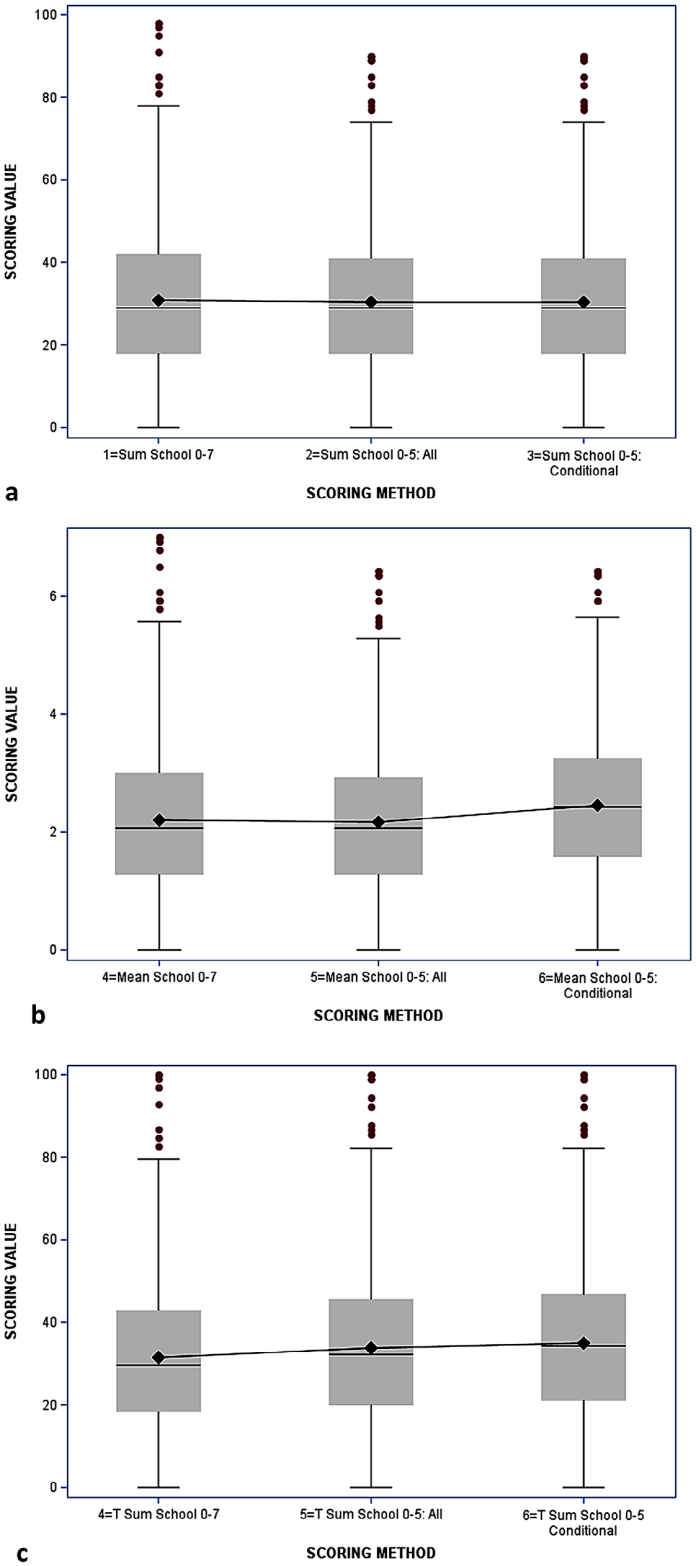
(**a**) (simple sum) (**b**) (mean scoring) (**c**) (transformed scoring): Means for different scoring methods for the EASY instrument using box and whisker plots. The bars represent the upper and lower confidence limits with the dots representing the outliers for each analysis. The line in the box is the median and the diamond is the mean with the line connecting the 3 box plots demonstrating the change by condition

A summary of the 3 scoring methods shows that there is no statistically significant difference across the scoring methods by grade groups (Table 3; Fig. 1. A paired t-test evaluation of the original scoring methodology to both the 0–5 and 0–5 conditional methods show that while the scores are very similar within methodology (summation, mean, transformed sum) they are all significantly different from one another, which demonstrates that for any given individual the scoring methodology used can result in meaningful differences. An ANOVA analysis evaluated three direct effects: the scoring method (*p* = 0.001) was statistically significant, the range of school items (0–7 days vs. 0–5 days) and the conditional use of the school items were not.

**Table 3:** Detailed data for different scoring methods for the EASY instrument (*N* = 614)

Scoring method	School items range	Inclusion of school items	Minimum	Maximum	Mean	STD*	t-test
**Simple summation**	0–7	All	0	98	30.86	17.53	
	0–5	All	0	90	30.4	16.89	0.01
	0–5	Conditional	0	90	30.4	16.89	0.01
**Mean**	0–7	All	0	7	2.2	1.25	
	0–5	All	0	6.43	2.17	1.21	0.01
	0–5	Conditional	0	6.43	2.45	1.22	0.01
**Transformed sum**	0–7	All	0	100	31.49	17.89	
	0–5	All	0	100	33.77	18.76	0.01
	0–5	Conditional	0	100	34.93	18.45	0.014

*STD = standard deviation

**Fig. 2:**
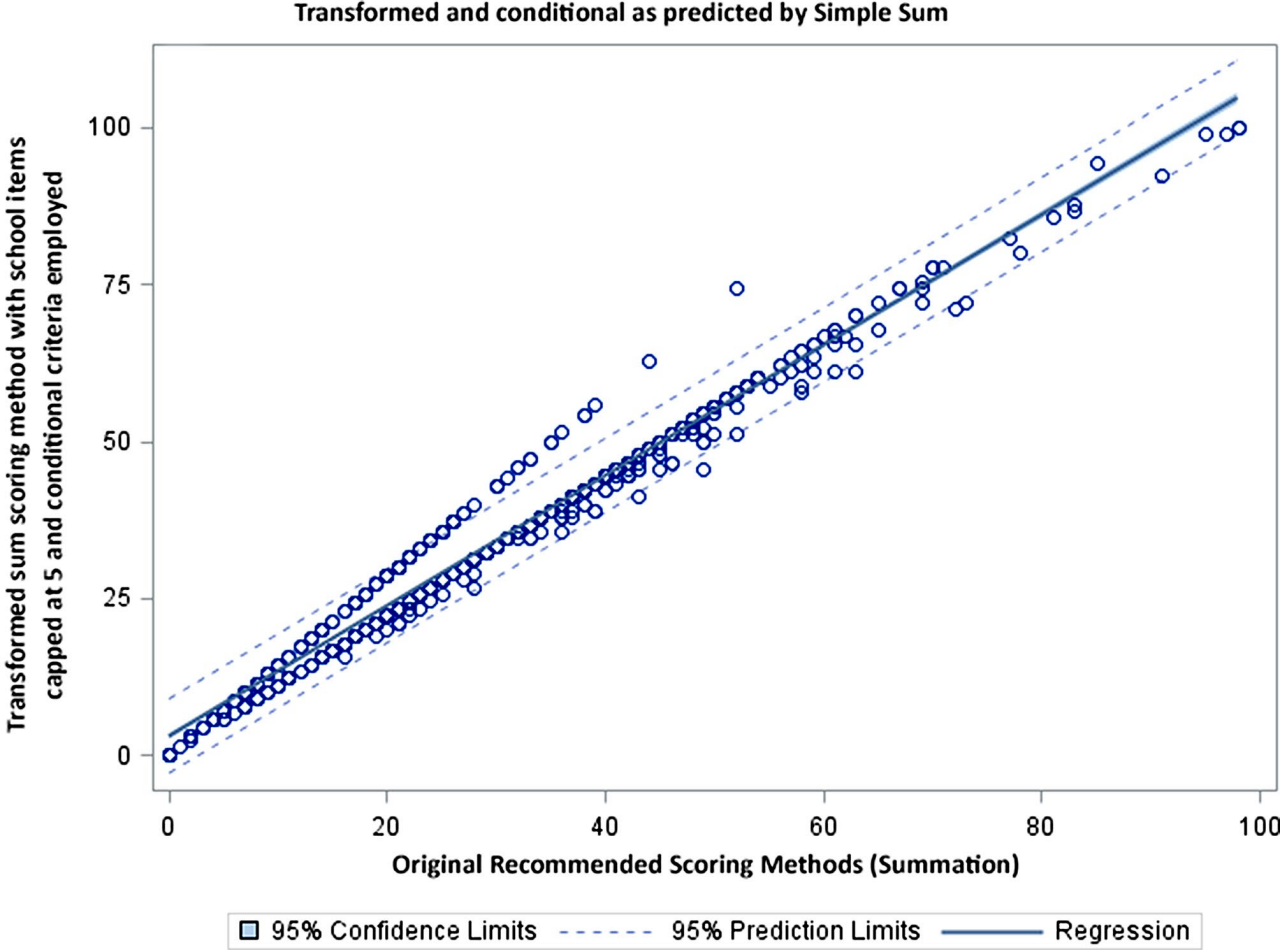
Graphic compares the recommend scoring with the transformed sum; while many cases fall on the regression line (Rsq 0.98), there is a distinct group that deviates and have a higher activity level when the transformed sum is used and a large number in which the scoring is slightly lower

**Fig. 3:**
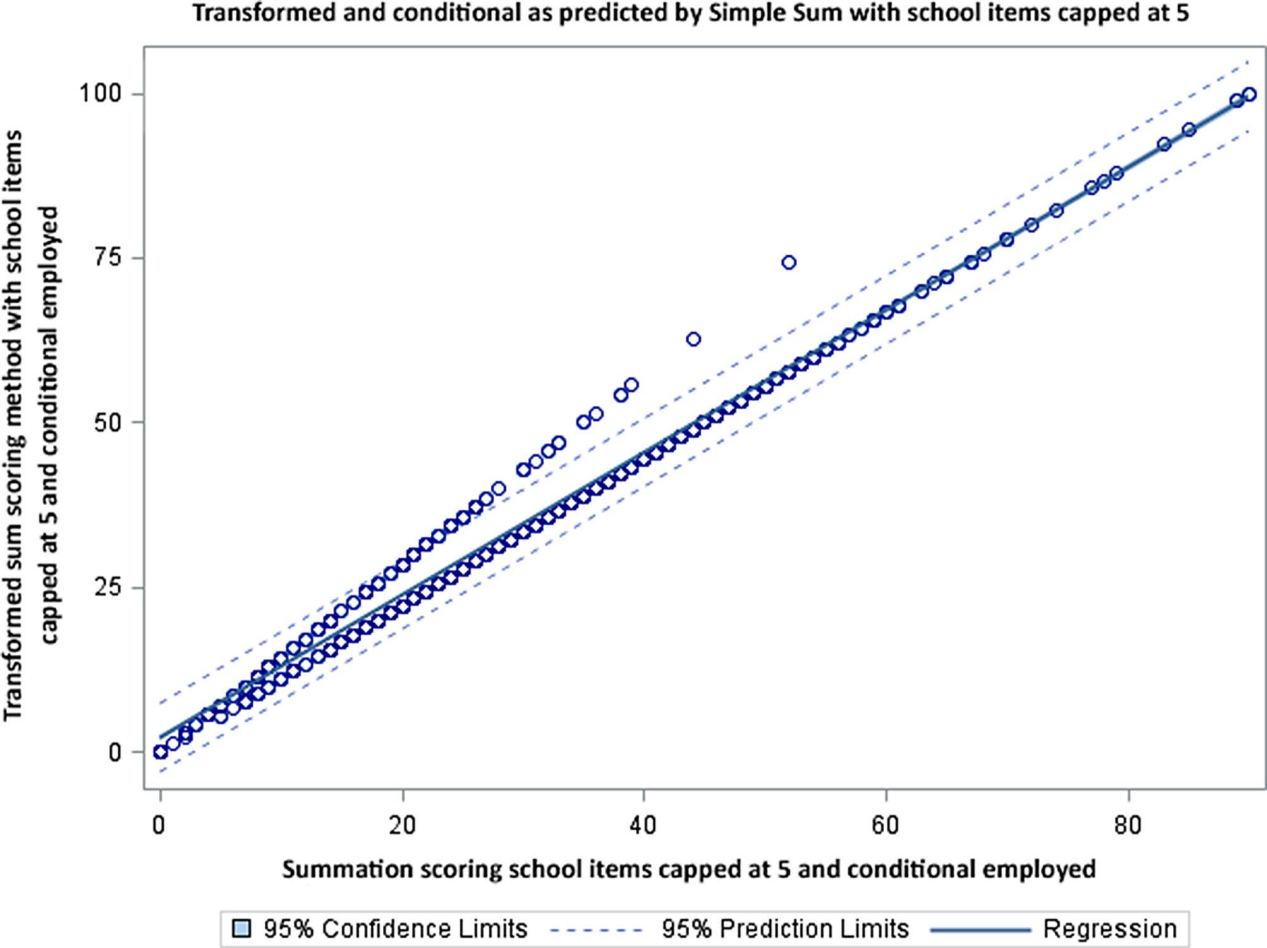
Graphic demonstrates a similar pattern (school items capped at 5, conditional scoring) in which there is a distinct group with higher scores, but no scores which are substantially lower

**Fig. 4:**
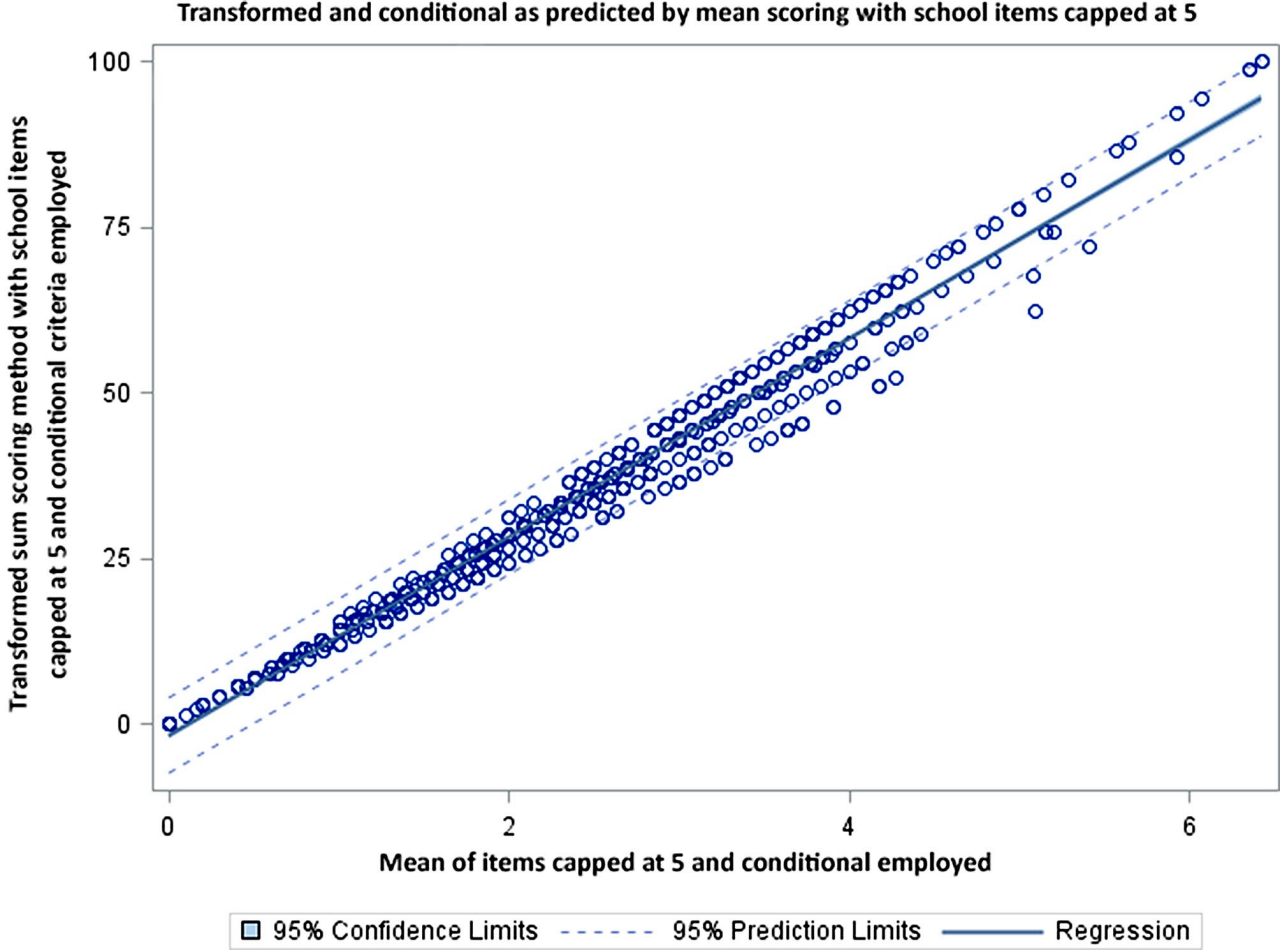
Graphic is the mean scoring method and it shows a different pattern in which low scores are fairly uniform, but as scores increase the number of cases diverging above and below increases as well

Figures 2, 3 and 4 compares the scoring options of simple and mean scoring to the transformed score with school items capped at 5 and conditional criteria employed. Figure 2 evaluates the original scoring methodology, Fig. 3 evaluates the simple summation with the school items capped at 5 and Fig. 4 evaluates the mean score with the school items capped at 5. All analyses result in R-squared of 0.97 or 0.98. The dispersion of the scores around the regression line demonstrate the magnitude of under or over estimation of the individual scores.

Figure 2 shows that there is one group that is systematically different. The simple summation method of scoring over-estimates the reported physical activity level (demonstrated by the circles above the dotted line).

Figure 3 demonstrates the difference between capping school related items at 5 and not capping school related items at 5 but allowing them to reflect 7 days a week. This shows that bias still exists.

Comparison of the results for grades 1–5 (recommended sample) and those in grades 6–8 (original sample) indicated the survey was appropriate for both age groups with chi-square demonstrating no statistically significant difference between the two populations.

## Discussion

To address concerns relative to the scoring of the EASY which was administered during what most would consider summer vacation from school, alternative scoring was evaluated. We evaluated whether the four school related items should have a range of 0–7 days or 0–5 days. As shown in Table 1 between 5 and 10% of the overall study population indicated that their child participated in these school related activities 6 or 7 days a week which we consider to be respondent error.

There is a strong tendency in health and public health research to rely on simple summation as a scoring method. In the instance of the EASY this is a prime concern; if a child is on break from school the maximum range for summation scoring is 70 if school related questions are eliminated, not 98 (as per the original scoring) or 90 if school items are capped to a maximum of 5 days a week, the common number of days of school. Other factors may include that not all schools have recess on a daily basis, and this may change by grade, some schools do not allow or make it feasible to walk or bike to school and this may be individual student or grade dependent within a school. While simple imputation methods such as mean replacement are traditionally used to account for incomplete data; the fundamental nature of missing the school items during school break times, requires a different scoring methodology or an additional question indicating if school is in session.

The transformed sum accounts for both missing data and differing ranges in calculating the final score. Transformed sums are calculated using the following method: ((∑answers-absolute minimum)/range)*100. This is a more complex scoring method in that the absolute minimum value and the range are both determined by the answers provided by any given respondent.

For each method the descriptive statistics associated with the scoring method are very similar regardless of grade and whether the 0–7 or 0–5 range is used. The paired evaluation of the ranges demonstrate that the scoring method does significantly alter the responses of enough individuals that the t-test is significant.

While the R-squared across all models are in the 0.97-0.99 range there remains a systematic under or over estimation of activity across the 3 methods [10]. In all analyses there is an apparent group of individuals that are outside the 95 CI level of activity. This concern is easily seen in the plots (Figs. 2, 3 and 4) where there is notable dispersion in the scatter. The simple summation shows the number of children for whom the score will be underestimated (above the regression line) or overestimated (below the regression line). In Figs. 3 and 4 we get remove the overestimated activity cases by capping the days and still retain those being under-estimated (above the regression line). For those scores below the line the simple summation will give them a higher activity level than the transformed sum. The analyses provided here demonstrate that the scoring method used simple summation, mean, and transformed sum will significantly alter scores.

The original article references the use of 22 as a score cut-off for physical activity. Further work will need to be undertaken to find comparable cut-offs depending on the scoring method preferred by the researcher.

There is generalizability to this data because it is a scoring measurement evaluation of a survey. It was tested on the original study population it was designed for as well as the developers recommended expanded population of children. We did not find differences in response pattern within either sample of respondents to the survey from our population. We believe that the findings of our scoring recommendations which addresses scoring measurement methodology will hold true for any population of children. The scoring results are different based on methodology employed and should be considered by users of the EASY. The limitation to our quota sampling may be the magnitude of the differences in scores found across methods and conditions.

## Conclusion

We have demonstrated different scoring options and their impact on the final score to address the concerns that arose from our own dataset. A review of scoring options indicated that the transformed sum provided the strongest methodologic result. Our analysis also concluded that administering the scale by proxy to children from grades 1–8 resulted in similar responses broadening the potential use of this scale.

We propose that future researchers consider using a transformed sum for scoring and add an additional item with the instrument: (Are you currently in school?) capping the maximum range of days per week for the school related activity at 5.

## Data Availability

The datasets generated and/or analyzed during the current study are not publicly available but are available from the corresponding author on reasonable request.
